# Operationalizing Large Language Models for Clinical Research Data Extraction: Methods, Quality Control, and Governance

**DOI:** 10.1007/s10916-026-02353-w

**Published:** 2026-02-25

**Authors:** Lin Chen, Rui He, Puxuan Lu, Ying Jin, Li Zhou, Ning Li, Pengliang Wu, Bosen Hu

**Affiliations:** 1 Shenzhen Center for Chronic Disease Control, Shenzhen, 518020 China; 2 Cold Lake Prism Light Technology (Wuhan) Co., Ltd, Wuhan, 430001 China; 3 Dapuqiao Community Health Services Center, Huangpu District, Shanghai, 200023 China; 4 Department of Respiratory and Critical Care Medicine, Ruijin Hospital, Shanghai Jiao Tong University School of Medicine, Shanghai, 200025 China; 5 Department of Preventive Medicine and Health Care, Department of Information Technology, Dapuqiao Community Health Services Center, No. 712 Liyuan Road, Huangpu District, Shanghai, 200023 China

**Keywords:** Large language models (LLMs), electronic medical records (EMRs), information extraction, natural language processing, structured data

## Abstract

Methods

This narrative review drew on targeted searches of PubMed/MEDLINE and arXiv (January 2020–October 2025), verification of peer-reviewed versions via ACL Anthology for selected preprints, and citation tracking of seminal literature. In this review, we trace the methodological evolution from rules to encoder-based models and LLMs, propose a multidimensional evaluation framework for real-world deployment—which includes accuracy, structural quality, human-in-the-loop effort, stability, and compliance—and develop an operational governance checklist to support auditable and reproducible implementations. Using representative tasks—diagnosis extraction, medication records, clinical trial data, and phenotype integration—we summarize the improvements and failure modes of LLM-based extraction and analyze key challenges, including domain shift, factual “hallucinations,” privacy and regulatory constraints, and cost/latency trade-offs. Finally, we outline future directions through which multimodal and cross-lingual extensions, human–machine collaborative annotation, and standardized reporting practices can advance precision medicine and sustainable, high-quality clinical research.

**Supplementary Information:**

The online version contains supplementary material available at 10.1007/s10916-026-02353-w.

In healthcare research, key variables are often manually extracted from free-text electronic medical record (EMR) systems. Common tasks include identifying and characterizing thyroid nodules in ultrasound reports, or locating and recording the maximal carotid intima-media thickness (IMT) for analysis. Although such information is highly valuable for epidemiological research, quality improvement, and clinical decision support, the heterogeneity of phrasing, inconsistent formatting, and cross-sentence dependencies often prevent reliable, automated conversion into analysis-ready structured data. Consequently, many projects remain dependent on labor-intensive manual chart review. [1]

Traditional approaches, such as keyword search and rule-based regular expressions, perform well in narrowly scoped settings with rigid documentation templates. However, they struggle with negation and uncertainty, temporal reasoning, and cross-paragraph dependencies, as well as variation in writing conventions across institutions and clinical services. Consequently, precision and portability often decrease, maintenance costs increase and reusability decreases, thus creating a persistent “last-mile” bottleneck in the preparation of research-grade datasets. [1, 2] In recent years, large language models (LLMs)—fortified by pretraining at scale and instruction alignment—and their integration with retrieval augmentation and structured output constraints have enabled a prompt-driven, “generation-as-structured-output” extraction paradigm. With limited additional annotation in some settings, free text may be transformed into back-fillable, auditable fields and code mappings, thus opening a practical path to high-throughput curation and automated backfilling. [3] Notwithstanding these advances, domain shift, factual consistency (i.e., hallucination), privacy compliance, and operational reproducibility remain critical constraints that must be explicitly managed.

Building upon the above use cases and methodological progress, in this review, (i) the evolution from rule-based systems and encoder-style pretraining to LLMs is traced; (ii) benefits and failure modes are synthesized across representative tasks—diagnosis extraction, medication records, clinical trials, and phenotype integration; and (iii) a multidimensional evaluation and reporting checklist for clinical-grade deployment, together with practical considerations for privacy and engineering governance, is proposed, with the aim of providing an actionable framework for clinical and research teams.

## Methods

We conducted a narrative review with targeted searches and citation tracking. We searched PubMed/MEDLINE and arXiv for records published from 1 January 2020 to 31 October 2025. The searches were initially run up to 31 October 2025 and were updated during revision to include studies published up to 07 February 2026. The PubMed search combined keyword blocks related to large language models (e.g., “large language model”, LLM, GPT, ChatGPT), clinical text sources (EMR/EHR/clinical notes), and information extraction (e.g., information extraction, named entity recognition), with additional terms for structured outputs (e.g., schema-/JSON-constrained generation), retrieval-augmented generation (RAG), and evaluation or governance. For selected arXiv preprints, we cross-referenced ACL Anthology and journal databases to identify corresponding peer-reviewed or camera-ready versions when available. To ensure coverage of foundational concepts and benchmarks, we additionally performed backward and forward citation tracking and included seminal works published prior to 2020 when they were central to the topic.

Studies were included if they met the following criteria: (1) focused on clinical EMR text extraction using LLMs or LLM-centered pipelines; (2) involved structured output generation (e.g., JSON or schema-constrained outputs); (3) reported quantitative performance metrics (e.g., F1 score, exact match [EM], Cohen’s κ); (4) were conducted on real-world clinical datasets or well-described benchmarks. We excluded studies that primarily addressed conversational or advisory use cases without extraction tasks, non-clinical text mining applications, opinion or commentary articles without methodological detail, and duplicate or superseded preprints.

### Study Selection and Transparency

Records retrieved from PubMed/MEDLINE and arXiv were screened by title and abstract for relevance, followed by full-text assessment when necessary. Duplicate records and superseded versions were removed prior to synthesis. The overall identification and selection process is summarized in Supplementary Figure S1 (Narrative review with targeted searches and citation tracking).

### Evidence-Tier Classification

Given the heterogeneity of study designs and evaluation settings, we adopted a pragmatic evidence-tier classification rather than formal risk-of-bias scoring. Greater interpretive weight was assigned to studies reporting real-world clinical data, external validation, and structured evaluation metrics. This approach was intended to enhance transparency while remaining consistent with the narrative scope of the review.

Structured extraction from clinical text: Traditional approaches, pretrained encoder models, and LLMs.

**Fig. 1:**
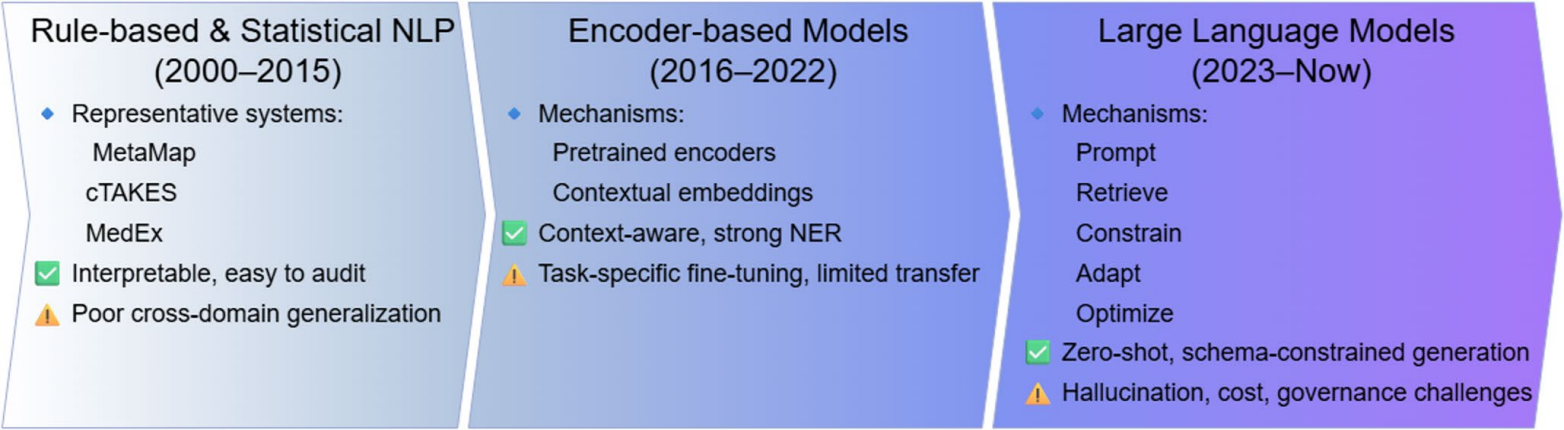
Evolution of Clinical Information Extraction. Rule-based and statistical systems (2000–2015) provide interpretable but domain-specific performance. Encoder-based models (2016–2022) improve contextual understanding but require task-specific fine-tuning. Modern LLMs (2023–present) enable zero-shot and schema-constrained generation while introducing new governance and cost challenges

### Rule-Based and Statistical Learning

Structured extraction from clinical text has evolved across multiple methodological eras (Fig. 1), beginning with rule-based, lexicon-driven systems and progressing to statistical and neural approaches. Early rule-based systems relied on curated dictionaries and regular-expression templates, offering strong interpretability but limited adaptability to stylistic variation and cross-institutional deployment. [4–7] Sequence-labeling models such as conditional random fields and support vector machines reframed named entity recognition (NER) as a supervised tagging task, improving boundary detection but remaining dependent on annotated corpora and domain-specific tuning. [8–11] Subsequent deep learning approaches introduced contextual representations that improved performance on NER and relation extraction; however, they remained sensitive to annotation size, class imbalance, and limited long-range reasoning capacity. [12, 13] Encoder-based transformer models further enhanced contextual modeling through pretraining and fine-tuning, [14–17] yet they largely preserved a task-specialized paradigm requiring separate models for each task, with limited zero-/few-shot transfer and constrained support for consistent structured outputs across long clinical documents. [18] These limitations collectively motivated the shift toward generative large language models capable of cross-task generalization and structured output generation.

### From Pretrained encoders to the LLM Paradigm

The rise of LLMs is grounded in statistical learning and early neural architectures and reflects substantial advances in natural language processing (NLP). Recurrent neural networks (RNNs) enable models to capture sequential dependencies and contextual information more effectively. [19] In contrast, the transformer architecture—through its self-attention mechanism and highly parallelizable training—substantially improves the modeling of long-range dependencies and training efficiency and has catalyzed the shift to a generative paradigm that is now dominated by LLMs. [20–22] Representative LLMs include OpenAI’s GPT-3.5/4 (the ChatGPT family) and Google’s PaLM 2; these systems demonstrate strong capabilities in terms of language understanding, generation, and cross-task transfer, with applications that extend from general text processing to medical text analysis and clinical decision support. [20, 21, 23] In addition to strong semantic understanding and generation, methodological advances also include large-scale pretraining, which equips models with broad, generalizable linguistic patterns; [14, 21] instruction alignment, such as instruction tuning and preference alignment, which improves controllability and adherence to task specifications; [3, 23] and retrieval-augmented generation (RAG) and structural output constraints (function calling/JSON schema), which may improve factual errors and inconsistencies in generated outputs. [24] LLMs can handle long-range dependencies across sentences and sections even in the absence of hand-crafted rules, which confers clear advantages for information extraction from clinical free-text narratives. [20, 25, 26]

### LLM Extraction Mechanisms: Prompting, RAG, Schema Constraints, PEFT, and APO

To convert clinical free text into machine-readable structured data, an LLM-centered workflow has emerged in which several components operate in a coordinated manner. Prompting (zero-/few-shot) makes task objectives and output formats explicit, standardizing label definitions and field constraints. Retrieval-augmented generation (RAG) introduces versioned authoritative resources—such as guideline excerpts, ontologies, and institutional lexicons—at inference time to enhance factual grounding. Schema-constrained generation (e.g., JSON schema or function calling) prespecifies field types and validation rules, enabling “generation-as-structuring” with database-ready outputs. Domain adaptation aligns local terminology and documentation styles under limited data and computing resources. Parameter-efficient fine-tuning (PEFT), particularly low-rank adaptation (LoRA), allows task alignment while minimally updating base model weights. Continued pretraining strategies such as domain-adaptive pretraining (DAPT) further strengthen domain representations. Finally, automatic prompt optimization (APO) supports version-controlled template libraries and systematic regression testing. [22, 24, 27–31]

In practice, structured extraction pipelines typically incorporate deterministic decoding and predefined output schemas to ensure reproducibility and programmatic validation. Broader issues related to evaluation, drift control, privacy, and governance are discussed in subsequent Sects. [27, 28, 32, 33]

**Fig. 2:**
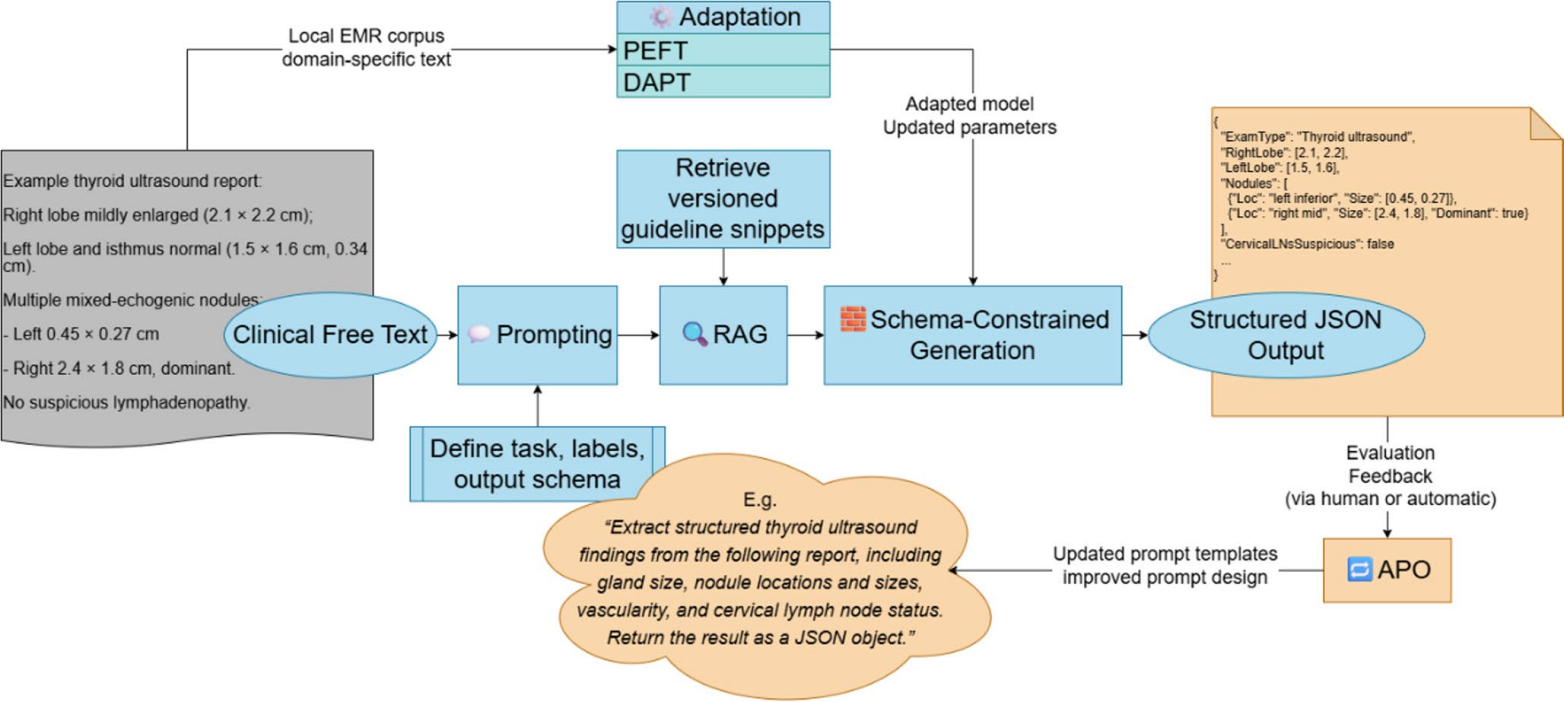
Prompt–Retrieve–Constrain–Adapt–Optimize (PRCAO) workflow for LLM-based EMR structuring**.** The pipeline integrates prompting, retrieval-augmented generation (RAG), schema-constrained output, and adaptive optimization strategies (PEFT, DAPT, APO) to convert example ultrasound reports into version-controlled JSON structures with feedback-driven refinement

**Table 1:** Three-Layer Operational Framework for LLM-Based EMR Structuring (PRCAO–Evaluation–Governance Model)

Dimension	Core Components	Principal Risks	Control Mechanisms	Key Metrics / Evidence
PRCAO Technical Generation Layer				
Prompt	Zero-/few-shot prompting; fixed output format; deterministic decoding	Prompt sensitivity; inconsistent field generation	Low-temperature decoding; fixed key order; versioned prompt templates	Task accuracy (F1/EM); cross-run consistency
Retrieve (RAG)	Versioned authoritative evidence; guideline excerpts; terminologies (ICD, SNOMED CT, LOINC, RxNorm)	Knowledge staleness; hallucination; OOD degradation	Version-tagged retrieval; source citation; evidence span return	Normalization accuracy; OOD validation
Constrain (Schema)	JSON schema; function calling; field constraints; “generation-as-structuring”	Parsing failure; field omission; semantic mismatch	Schema validation; JSON parsing checks; programmatic unit tests	PSR; FCR; semantic consistency
Adapt (PEFT/DAPT)	PEFT (LoRA); DAPT; institution-specific alignment	Overfitting; catastrophic forgetting; domain shift	On-prem adaptation; external validation; differential evaluation	External validation; drift analysis
Optimize (APO)	Automatic Prompt Optimization (APO); versioned template library	Prompt regression; maintenance burden	Regression test sets; change logs; differential testing	Stability across releases
Multidimensional Evaluation & Assurance Layer				
Accuracy	NER, relation extraction, code normalization	Overstated superiority; single-metric bias	Strict vs. lenient criteria; macro/micro reporting; bootstrap CIs	F1; EM; κ; confidence intervals
Structured-Output Quality	Backfillable outputs; database-ready JSON; EDC/CRF integration	Non-persistent outputs; code-mapping errors	PSR; FCR; source-span agreement	PSR; FCR; normalization accuracy
HITL Burden	Selective prediction; uncertainty-triggered review	Automation bias; reviewer fatigue	Coverage–accuracy curves; abstention thresholds; adjudication workflow	Review time; correction rate; κ
Stability & Calibration	Run-to-run consistency; version drift monitoring	Prompt/schema drift; calibration failure	Fixed decoding; regression suite; ECE; Brier score	ECE; Brier; cross-run agreement
Cost & Latency	Token cost; p50/p95 latency; throughput	Long-document latency; cost escalation	Chunk–summarize; batching; caching	Latency (p50/p95); token cost
Governance, Provenance & Lifecycle Control Layer				
Provenance & Evidence Traceability	Supporting source passage return; character span alignment; provenance labeling	Unverifiable outputs; traceability gaps	Mandatory source-span return; version-tagged RAG assets; audit logs	Auditability; reproducibility
Privacy & Compliance	Deidentification; data minimization; RBAC; gateway allowlists	PHI leakage; regulatory violation	On-prem inference; encrypted transport; DPIA; differential privacy (when appropriate)	Compliance documentation
Versioning & Drift Control	Prompt/schema/model/RAG version pinning	Output drift; silent regression	Replay & rollback; staged/canary release; differential evaluation	Drift reports; regression logs
Lifecycle Governance	Audit trails; change logs; service-level objectives	Loss of reproducibility; accountability gaps	End-to-end logging; controlled release cycles; accountability mechanisms	Traceable release history

The overall technical workflow of LLM-based structured extraction is illustrated in Fig. 2. This “prompt–retrieve–constrain–adapt–optimize” (PRCAO) paradigm provides a structured approach to addressing key challenges of structuring clinical text at the generation level. As further synthesized in Table 1, sustained and robust operation in real-world clinical research settings additionally requires a multidimensional evaluation panel and lifecycle-level governance, including provenance tracking, version control, and compliance safeguards. Illustrative institutional workflow for clinical-research deployment (example). To operationalize the governance controls in Table 1, an institution can implement a gated “extract–validate–review–release” pathway. A data steward (or clinical informatics lead) maintains a versioned schema/prompt library and a frozen, auditable retrieval bundle; each run logs the model/prompt/schema/RAG versions, decoding settings, input identifiers, validation outcomes, and any human edits. The pipeline first enforces programmatic checks (schema validation, field completeness, range/logic rules) and then routes cases to human adjudication when triggers fire (e.g., validation failure, low confidence/abstention, out-of-domain flags, or drift alarms). A reviewer adjudicates with provenance highlighting and records corrections, which are stored as structured patches linked to the original evidence spans. Releases are promoted only after regression tests on a retained set and a canary period; any update to prompts/schemas/models/RAG assets requires change logs, differential evaluation, and the ability to replay and rollback to a previous pinned version. Privacy and compliance oversight (e.g., IRB/data-protection review, RBAC, secure enclaves, and audit trails) is enforced throughout as part of the total lifecycle.

## Model Performance Evaluation and Assessment Framework

For structured extraction from clinical free text, point estimates of “accuracy” alone no longer capture real-world utility. Evaluation is shifting from single-number accuracy to a multidimensional composite: a panel that includes task accuracy, structured-output quality, human-in-the-loop (HITL) burden, stability/reliability, and regulatory compliance. In addition to this panel, cost-aware and selective prediction strategies dynamically allocate human review on the basis of predicted risk and uncertainty, and broader deployment considerations, such as fairness across patient subgroups and clinical services, should also be examined. Together, these elements constitute an integrated quantitative framework for clinical-grade deployment and lifecycle maintenance. [34, 35]

### Performance

Named entity recognition and relation/event extraction are typically evaluated using precision, recall, and F1 score, with explicit reporting of matching criteria (strict/exact vs. lenient/partial) and the level of aggregation (span level vs. document level). [36–40] For multiclass settings, we recommend reporting both macro- and microaveraged metrics, along with a subtask-level breakdown for entities, relations, and events. [41, 42] For the extraction-plus-normalization pipeline, complementary metrics such as exact match (EM) or code-level accuracy should also be reported.

### Structured Output Quality

To gauge the real-world viability of “generation-as-structured output,” we recommend reporting the following metrics: the parsing success rate (PSR), namely, whether the output passes JSON schema or function-signature validation; the field completeness rate (FCR), which is the proportion of required fields that are nonempty and valid; the semantic consistency, which represents the agreement between structured values and the source span/evidence (via manual adjudication or explainable alignment scores); and the normalization accuracy, which represents the correctness of terminology/code mappings and the success rate of disambiguation. [43] These metrics are key determinants of whether outputs can persist in databases, backfilled, and ingested into electronic data capture (EDC) systems and case report forms (CRFs) and should be reported alongside conventional accuracy measures. [38, 44–48]

### Practical Utility and Cost

To quantify human–machine collaboration costs, we recommend reporting the manual review time (minutes per document), clinician acceptance rate, and rework rate. Performance and resource utilization should be summarized as inference latency (p50/p95), throughput (documents per minute), and compute/token cost (e.g., GPU hours per document or currency per 1,000 tokens). Collectively, these measures provide practical indicators of operational feasibility for in-hospital deployment and should be monitored longitudinally across model iterations. [34, 48–50]

### Stability and Reliability

Run-to-run consistency—e.g., Jaccard or agreement scores that are obtained by repeatedly processing the same input—and sensitivity to version drift are evaluated by quantifying differences before and after prompt or schema changes. Confidence and the abstention policy are calibrated using coverage–accuracy curves, the expected calibration error (ECE), and the Brier score so that uncertain cases are automatically routed to human review or safely declined. [32, 34, 48, 49, 51]

### Evaluation and Reporting Standards

To ensure comparability and traceability, reports should include both internal (in-distribution) and external (out-of-distribution) validation and disclose interannotator agreement (e.g., Cohen’s κ). Data partitioning and leakage safeguards must be specified (e.g., no patient or encounter appears across splits). Key metrics should be accompanied by confidence intervals (e.g., bootstrap 95% CIs) and stratified error analyses—such as buckets for negation, uncertainty, temporality, abbreviations, and other challenging cases. Decoding strategies and random seeds should be fixed and documented to ensure reproducibility of reported metrics. [32, 48, 52–54]

In summary, technical performance, structured-output quality, operational burden, and stability constitute the core evaluation framework for EMR extraction. Coupling this framework with cost-aware and selective prediction strategies enables quantitative go/no-go deployment decisions and sustainable monitoring across model iterations. Governance and lifecycle controls, including version pinning, audit logging, and compliance safeguards, are addressed in the subsequent sections.

## Applications of LLMs for Information Extraction From Electronic Medical Records

**Table 2:** Representative LLM-based EHR extraction systems comparison

Study	Task / use case	Setting & data (*N*)	Model / approach	Key results (reported)	Evidence tier
Chung et al[55]	Perioperative risk prediction (ASA-PS, admission/ICU, mortality, duration)	Retrospective EHR; 3 academic hospitals; 2y (single metro area)	GPT-4 Turbo; prompting variants incl. summaries/few-shot/CoT	Mortality F1 = 0.86; ICU admission F1 = 0.81; Hospital admission F1 = 0.64; ASA-PS F1 = 0.50; PACU MAE = 49 min	Tier B
Bürgisser et al[56]	Disease detection (gout, CPPD) from French EHR paragraphs	Geneva tertiary hospital; 700 gout + 600 CPPD paragraphs	Llama-3-8B; few-shot + CoT prompting	Gout: PPV 92.7%, NPV 96.6%, Acc 95.4%; CPPD Acc 94.1%	Tier B
Pan et al[57]	Disease labeling (AMI/diabetes/HTN) from EHR notes vs. ICD/clinician labels	Cardiac registry 2015; 3,088 pts; 551k notes; linked Alberta EHR	Mistral-7B-OpenOrca; guideline-anchored prompting	AMI: Sens 88%, Spec 63%, PPV 77%; Diabetes: Sens 91%, Spec 86%, PPV 71%	Tier B
Johnson et al[58]	Pathology phenotype extraction (colorectal dysplasia/HGD/CRC)	VHA pathology reports 1999–2024; 116,373 reports (MVP biobank)	Gemma-2; search-term filtering + yes/no prompts	IBD: dysplasia F1 96.9%, CRC F1 98%; non-IBD: dysplasia F1 99.2%, CRC F1 95%	Tier A
Chen et al[59]	Oncology inference from radiology reports (pancreatic cancer status/location/response)	203 de-identified reports; 164 pancreatic tumor pts	LLM comparison (GPT-4/3.5, Gemma-7B, Llama3-8B, Mistral-7B); prompt engineering	GPT-4 F1-micro 75.5%; Mistral-7B 68.6%; Llama3-8B 61.4%	Tier C
Ding et al[60]	Health event prediction (HF, HTN) from multi-modal EHR	MIMIC-III; 7,125 pts	LLM + CKLE predictive model; cross-modality KD + contrastive objectives	Max + 4.48% accuracy over baselines	Tier B
Choi et al[61]	Injury info extraction/classification from ED notes (mechanism/place/activity/intent/severity)	2 urban tertiary hospitals; derivation 36k / test 32k pts	Fine-tuned Llama-2 + instruction prompting (vs. BERT baselines)	Mechanism Acc 0.899; Intent Acc 0.972; outperformed BERT across tasks	Tier A
Zolnoori et al[62]	MCI-ED screening (multimodal: speech + EHR notes + OASIS)	Ongoing VNS Health study; 114 HHC pts (55 cases/59 controls)	Schema-guided LLM pipeline + supervised ML integration	Multimodal > single-source; data collection feasible	Tier C

*ASA-PS* American Society of Anesthesiologists Physical Status, *ICU* intensive care unit, *PACU* post-anesthesia care unit, *AMI* acute myocardial infarction, *CPPD* calcium pyrophosphate deposition disease, *HF* heart failure, *HTN* hypertension, *VHA* Veterans Health Administration, *HGD* high-grade dysplasia, *CRC* colorectal cancer, *MCI-ED* mild cognitive impairment–early dementia, *HHC* home health care, *KD* knowledge distillation, *MAE* mean absolute error, *PPV/NPV* positive/negative predictive value

Evidence-tier definition: *Tier A* large-scale real-world data with clear held-out evaluation and stronger generalizability signals (e.g., multi-site and/or large external-like test, robust performance reporting, feasibility for deployment discussed), *Tier B* retrospective/single setting with clear test evaluation and quantitative metrics but limited external generalization and/or deployment detail, *Tier C* small-sample/pilot/protocol/feasibility or limited validation, mainly hypothesis-generating

Synthesis of cross-study trends (from Table 2 and Supplementary Table S1). Across the representative systems summarized in Table 2 (see detailed characteristics in Table S1), the dominant task formulation is classification or risk prediction (e.g., disease labeling, phenotype identification, and outcome prediction), often implemented as binary/ordinal decisions or categorical labels rather than fully structured, span-level extraction. A smaller subset moves closer to structured extraction, typically by constraining outputs (e.g., schema-guided or form-like outputs) and/or integrating additional modalities, whereas explicit normalization to terminologies/codes (with code-level accuracy reported) remains comparatively less represented in the applied end-to-end pipelines. Methodologically, prompting-centric workflows (often with few-shot and chain-of-thought variants) are prevalent, with emerging patterns that combine lightweight filtering, instruction-style prompting, or task-specific fine-tuning; multimodal fusion is beginning to appear but remains early. From an evidence perspective, most studies fall into Tier B (4/8), reflecting retrospective evaluation in a single setting with quantitative test metrics but limited external generalization and/or operational detail; Tier A evidence (2/8) is less common and is mainly seen in larger-scale, deployment-oriented evaluations; Tier C (2/8) consists of pilots or feasibility-focused work. Overall, the distribution highlights a recurring gap between benchmark-like performance reporting and generalizable, real-world validation, reinforcing the need for external evaluation, operational reporting (e.g., stability/cost), and auditable governance when translating extraction systems into clinical research workflows.

### Automated identification of diagnoses

Diagnostic information is among the most critical components of the EMR. Automating its extraction can substantially improve the efficiency and accuracy of clinical data processing, thereby enabling clinicians to rapidly assess a patient’s condition and formulate management plans. By integrating NLP with structure-aware parsing, key elements can be distilled from large volumes of heterogeneous clinical narratives, including disease names, diagnostic statements, and descriptions of disease status. [39, 43, 63]

LLMs can automatically recognize diagnostic terminology and related concepts within narrative text and convert them into structured data that are suitable for clinical decision-making. In practice, augmenting this process with deep-learning-based text annotation and named entity recognition substantially improves the accuracy of diagnostic information extraction. [39, 40]

### Structured Processing of Medication Orders

Medication orders constitute a core component of EMRs, and each order captures not only the drug name but also the dosage, frequency, and route of administration. Converting these elements into structured data via computational pipelines is critical for prescription management, medication safety surveillance, and downstream pharmacoepidemiologic analyses. [64] By leveraging NLP, the errors that are introduced by manual data entry can be effectively reduced, and the accuracy and efficiency of information extraction can be improved.

In practice, LLMs can automatically identify drug names, dosages, and administration instructions from prescription text and, via structured parsing utilities, convert the extracted elements into standardized representations, thereby facilitating downstream review and aggregation. In a study of prescription records, a pipeline that combines NLP with structured parsing achieved high performance for medication extraction, with an overall accuracy of approximately 89.5% and a recall of approximately 85.3%. [65] This approach markedly improves the completeness and internal consistency of prescription records, thus providing a more reliable foundation for clinical analytics and medication-related research.

### Clinical Trial Data Extraction

The extraction and curation of clinical trial data are pivotal for biomedical research. The large-scale deployment of LLMs now makes retrieving trial-relevant variables directly from EMRs feasible, thereby supporting clinical research and drug development. [66, 67] By implementing an EDC system that is anchored in structured CRFs, databases can be instantiated automatically, and trial data can be processed efficiently. [47] Next-generation EDC platforms increasingly integrate LLMs to parse complex trial data, such as patient clinical variables, treatment regimens, and outcome measures. This approach not only accelerates data extraction but also increases accuracy and consistency; moreover, the automation can be tailored to trial-specific requirements to support customized data curation and analysis. [68] Thus, LLMs can markedly increase the efficiency of converting EMR content into analysis-ready data for real-world research applications, thus providing clinicians with new tools while accelerating progress in medical research and drug development.

### Phenotyping and Multimodal Data Integration

Building on robust text-to-phenotype extraction, several studies have demonstrated end-to-end multimodal integration that links structured variables from narrative reports with imaging, genomics, and longitudinal EHR tables. In oncology, a large retrospective framework that integrates chest computed tomography (CT), chief-complaint narratives, laboratory values, and demographics to predict EGFR mutation status and survival was developed, and it achieved strong area under the curve (AUC) values with external validation and resilience to missing modalities. [69] Complementarily, comparative evaluations have shown that general-purpose LLMs can synthesize multimodality breast imaging reports (mammography, ultrasound, magnetic resonance imaging (MRI), and positron emission tomography (PET)/CT) into structured summaries with high accuracy for lesion characteristics, metastatic assessment, and TNM staging, alongside favorable clinician satisfaction. [70] At the system level, a continuous multimodal data-supply chain has been operationalized in a cancer center to integrate real-time clinical, molecular, and imaging data; ETL pipelines with NLP transform unstructured notes into analysis-ready tables under extensive quality-control rules, thereby enabling survival analyses and decision support within a governed, auditable workflow. [71] Together, these advances position text-derived phenotypes as anchors for cross-modal linkage—via accession/encounter identifiers and standardized terminologies—while highlighting the importance of validation across institutions and sustained data engineering governance for scalable clinical deployment.

## Challenges in Structured Information Extraction

While technical mechanisms and governance safeguards are described above, real-world deployment exposes residual system-level risks that persist despite mitigation strategies.

### Algorithmic Dimension: Semantic Ambiguity, Knowledge Staleness, and Hallucinations

Clinical narratives frequently contain negation, uncertainty, and cross-sentence dependencies, while medical knowledge and terminologies evolve rapidly. These factors increase susceptibility to distorted cross-span reasoning, outdated knowledge use, and factual hallucinations, thereby threatening the reliability of code mapping and downstream statistical inference. [15, 21, 72, 73] Although mechanisms such as RAG, schema-constrained generation, selective prediction, and lightweight domain adaptation can mitigate these risks, algorithmic robustness remains sensitive to out-of-distribution (OOD) inputs and evolving institutional language.

### Data Dimension: Terminological Synonymy, Format Heterogeneity, and Annotation Scarcity

Clinical documentation exhibits terminological synonymy, heterogeneous formats, and limited high-quality annotations. Variability across institutions and departments complicates terminology normalization and structured field harmonization. These data-level challenges constrain generalizability and increase labeling costs. Although terminology mapping, schema alignment, weak supervision, and external validation can alleviate these issues, sustainable performance depends on consistent data governance and cross-institution evaluation.

### Ethical Considerations: Deidentification, Data Minimization, and Traceability

Because EMRs contain sensitive protected health information (PHI), strict adherence to deidentification, data minimization, and regulatory compliance is essential. [74, 75] Cross-border data transfer and cloud deployment require careful evaluation within institutional and jurisdictional frameworks. Governance safeguards—including controlled access, secure computation environments, and traceable audit mechanisms—are necessary to ensure accountability and reproducibility in clinical-grade deployments.

### Engineering Considerations: Versioning, Drift, and Reproducibility

Sustained deployment requires systematic management of versioning, drift, latency, and cost. Even minor updates to prompts, schemas, RAG resources, or model parameters may introduce output instability. Long-document reasoning further imposes latency and compute constraints. Therefore, structured regression evaluation, drift monitoring, and resource-aware processing are critical to maintaining reproducibility and operational feasibility over time. [75]

## Future Directions

Future work should prioritize the standardization of evaluation and reporting frameworks for clinical-grade LLM deployment. Rather than relying on single accuracy metrics, research should focus on harmonized, multidimensional benchmarks that integrate structured-output validity, calibration, cross-institution generalizability, and cost-aware feasibility. Establishing community-wide reporting guidelines and shared regression datasets is likely to be necessary for enabling comparability and reproducibility across institutions.

Future research should also explore multimodal integration across text, imaging, physiologic waveforms, laboratory time series, and medication records on unified event timelines grounded in interoperable standards such as FHIR, with harmonized mappings to ICD, LOINC, SNOMED CT, and RxNorm. Cross-lingual and multilingual expansion remains critical for global deployment, requiring robust synonym alignment, transliteration strategies, and semantic equivalence testing. Evaluation protocols should incorporate cross-modal concordance and temporal consistency, as well as explicit out-of-domain (OOD) validation to avoid performance degradation beyond training institutions.

Future directions should emphasize scalable human–AI collaboration frameworks that balance automation efficiency with expert oversight. Iterative feedback loops, active learning strategies, and reusable annotation infrastructures will be important for sustaining data quality while reducing labeling burden across institutions.

Ethical and regulatory harmonization will remain a central priority. Future research should address cross-jurisdictional governance models, transparent risk stratification, and standardized auditability frameworks to support compliant deployment while preserving innovation. Emerging approaches such as federated learning and privacy-preserving computation warrant further evaluation under rigorous bias and fairness assessment.

Overall, LLMs have substantially advanced the structuring of free-text EMR data, offering a pathway toward standardized clinical research datasets and cumulative evidence generation. [76–79] However, domain shift, contextual ambiguity, factual hallucinations, and governance complexity remain persistent barriers to real-world adoption. Continued work on harmonized evaluation standards, integrated governance frameworks, and human-in-the-loop collaboration will be essential to achieving auditable, reproducible, and sustainable deployment in precision medicine and data-driven clinical research.

## Supplementary Information

Below is the link to the electronic supplementary material.

Supplementary Material 1

## Data Availability

No datasets were generated or analysed during the current study.
