# Supplementary material for: Operationalizing Large Language Models for Clinical Research Data Extraction: Methods, Quality Control, and Governance
Source: J Med Syst. 2026 Feb 25;50(1):25. doi: 10.1007/s10916-026-02353-w (PMC12932350; doi:10.1007/s10916-026-02353-w)
Supplement: Supplementary file 1 — Supplementary Material 1 [file 10916_2026_2353_MOESM1_ESM.docx]

**Supplementaries**


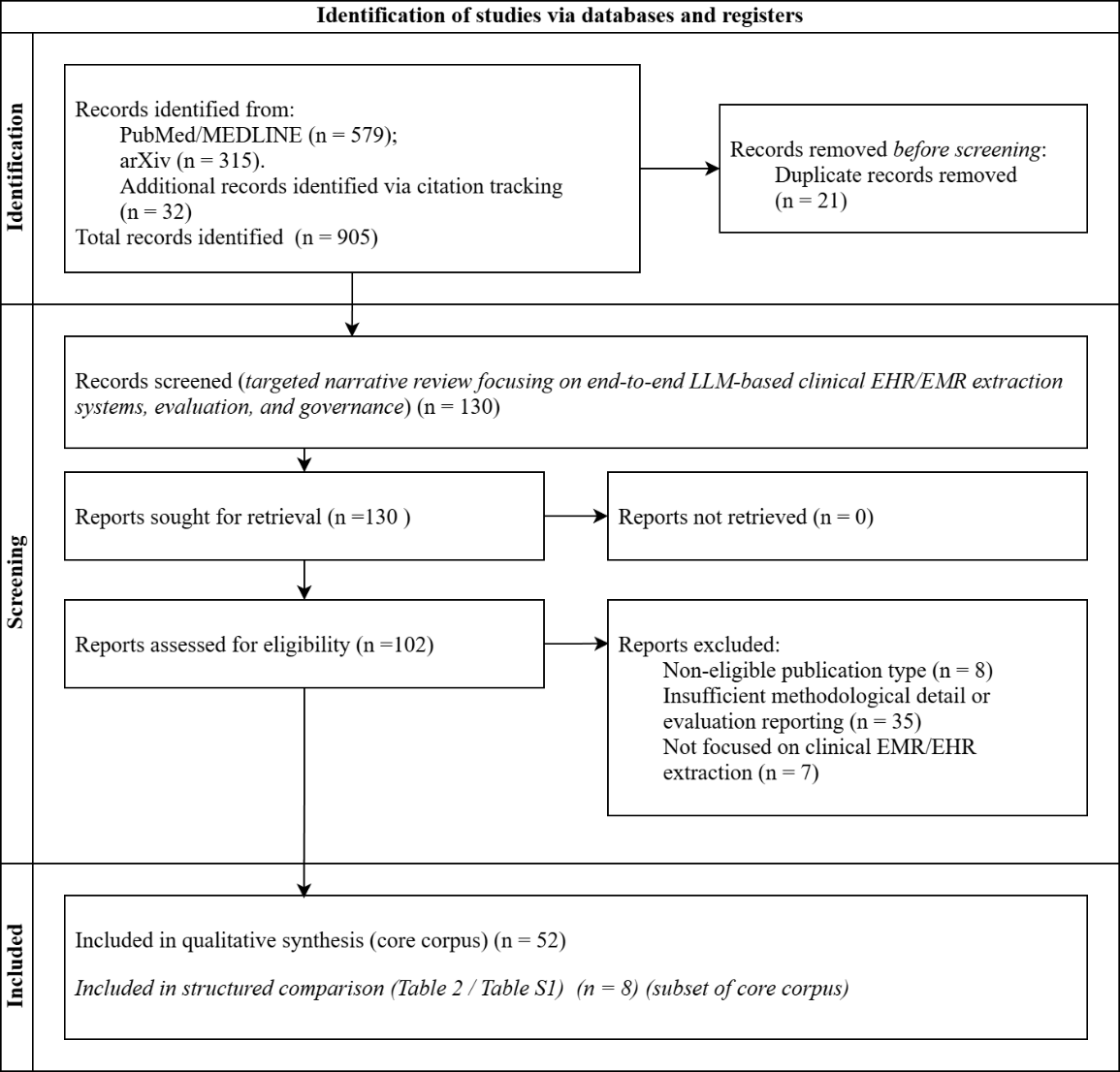


**Supplementary Figure S1. PRISMA-style flow diagram adapted for a narrative review with targeted searches and citation tracking.** Database searches identified 894 records (PubMed/MEDLINE n = 579; arXiv n = 315; via citation tracking n = 32), yielding 905 unique records after deduplication (duplicates removed n = 21). Citation tracking identified 32 additional records. A subset was prioritized for manual screening to focus on end-to-end LLM-based clinical EMR/EHR extraction pipelines with operational evaluation and governance considerations (n = 130). After screening, 52 studies formed the core qualitative synthesis, and 8 representative systems were included in the structured comparison (Table 2 and Table S1). The final reference list also includes background and contextual sources not counted as included studies in this flow.

**Supplementary Table S1. Detailed characteristics of studies included in Table 2.**

| **Author(s)** | **Task** | **Data source** | **Model** | **Pipeline components** | **Output form** | **Evaluation** | **Key results** | **Evidence tier** | **Deployment note** |
| --- | --- | --- | --- | --- | --- | --- | --- | --- | --- |
| Chung et al | Perioperative risk prediction (ASA-PS, hospital/ICU admission, mortality, duration outcomes) | 2y retrospective EHR from a quaternary care center (3 academic hospitals, single metro area) | GPT-4 Turbo | Prompting (original notes, summaries, few-shot, chain-of-thought) | Predictions + natural language explanations | F1 (binary/categorical), MAE (numerical duration) | ASA-PS F1=0.50; hospital admission F1=0.64; ICU admission F1=0.81; hospital mortality F1=0.86; PACU MAE=49 min | Tier B | NR |
| Bürgisser et al | Disease detection (gout, CPPD) from French EHRs | 700 manually reviewed gout paragraphs + 600 CPPD paragraphs from a Geneva tertiary hospital | Llama-3-8B | Few-shot prompting, chain-of-thought prompting | Disease/non-disease classification | PPV, NPV, accuracy | Gout: PPV=92.7%, NPV=96.6%, accuracy=95.4%; CPPD: accuracy=94.1% | Tier B | NR |
| Pan et al | Disease detection (AMI, diabetes, hypertension) from EHR notes | 2015 cardiac registry cohort (3,088 patients, 551k notes) linked to Alberta, Canada EHR | Mistral-7B-OpenOrca | Prompting based on diagnosis, treatment, clinical guidelines | Disease labels (vs ICD codes/clinician diagnoses) | Sensitivity, specificity, PPV | AMI: sens=88%, spec=63%, PPV=77%; diabetes: sens=91%, spec=86%, PPV=71% | Tier B | NR |
| Brian Johnson et al | Histopathologic diagnosis extraction (colorectal dysplasia, HGD/CRC, invasive CRC) from pathology reports | 116,373 VHA reports (1999–2024) from MVP biobank (IBD/non-IBD) | Gemma-2 | Search term filtering + "yes/no" question prompts | Diagnosis classification (yes/no phenotypes) | F1 scores | IBD: dysplasia F1=96.9%, CRC F1=98%; non-IBD: dysplasia F1=99.2%, CRC F1=95% | Tier A | Resource-limited computing environments (details NR) |
| Li-Ching Chen et al | Oncology data inference (pancreatic cancer presence, location, treatment response) from radiology reports | 203 deidentified radiology reports (164 pancreatic tumor patients) | GPT-4, GPT-3.5-turbo, Gemma-7B, Llama3-8B, Mistral-7B | Ablation, prompt engineering | Disease status, location, indeterminate nodules | F1-micro, precision, recall | GPT-4 F1-micro=75.5%; Mistral-7B=68.6%; Llama3-8B=61.4% | Tier C | NR |
| Sirui Ding et al | Health event prediction (heart failure, hypertension) from multi-modal EHR | MIMIC-III dataset (7,125 patients) | LLM + CKLE predictive model | Cross-modality knowledge distillation (KD), prompt learning, contrastive loss, patient similarity loss | Health event predictions | Accuracy (vs state-of-the-art) | Max 4.48% accuracy improvement over baselines | Tier B | NR |
| Dong Hyun Choi et al | Core injury info extraction (mechanism, place, activity, intent, severity) from ED notes | 2014–2020 retrospective data from 2 urban tertiary hospitals (derivation: 36k; test: 32k patients) | Llama-2 (fine-tuned), 5 BERT models | Fine-tuning, instruction prompting (task-specific) | Injury info classification (5 tasks) | Accuracy, macro-average F1 | Llama-2: mechanism accuracy=0.899, intent=0.972; outperformed BERT in all tasks | Tier A | Locally deployable (on-prem/cloud, logging, governance details NR) |
| Maryam Zolnoori et al | MCI-ED screening (multimodal: speech, EHR notes, OASIS) | Ongoing VNS Health study (114 HHC patients: 55 cases, 59 controls) | Schema-guided LLM pipeline (not specified) | Schema-guided extraction from notes/transcripts; supervised ML integration with speech/OASIS | MCI-ED classification | Feasibility, model performance | Preliminary: multimodal models outperform single-source; data collection feasible | Tier C | NR |
